# The phenomenon of desorption: What are the best adsorber exchange intervals?

**DOI:** 10.1186/s13054-024-04968-2

**Published:** 2024-05-27

**Authors:** Alix Buhlmann, Rolf Erlebach, Mattia Müller, Sascha David, for the BonHanZA study group, Eva-Maria Kleinert, Rolf Erlebach, Rea Andermatt, Daniel Andrea Hofmaenner, Mattia Mueller, Reto Schuepbach, Pedro David Wendel-Garcia, Christoph Camille Ganter, Klaus Stahl, Tobias Welte, Thorben Pape, Ann-Kathrin Rath, Bahar Nalbant, Jannik Ruwisch, Christian Bode, Benjamin Seeliger, Christian Putensen, Konrad Peukert, Andrea Sauer, Lennart Wild

**Affiliations:** Institute of Intensive Care Medicine, University Hospital Zurich, Rämistrasse 100, 8091 Zurich, Switzerland

**Keywords:** Clearance, Cytokine storm, Hemolysis, Removal, Saturation

## Comment

To the Editor,

With great interest we read the recent article in the *Journal* by Jansen et al. providing a proof-of-principle that treatment with CytoSorb^®^ hemoperfusion (HP) reduces plasma concentration of circulating cytokines in a model of systemic inflammation and that this reduction of circulating cytokines does not negatively affect the induction of endotoxin tolerance [1]. In this study, twenty-four healthy volunteers received an intravenous LPS challenge twice, half of which underwent HP during the first challenge and the cytokine response, as well as the degree of endotoxin tolerance were analyzed. The authors need to be congratulated for performing this study, providing important data that HP might be of clinical value for patients with hyperinflammation without having a late impact on immunological phenotypes.

**Fig. 1:**
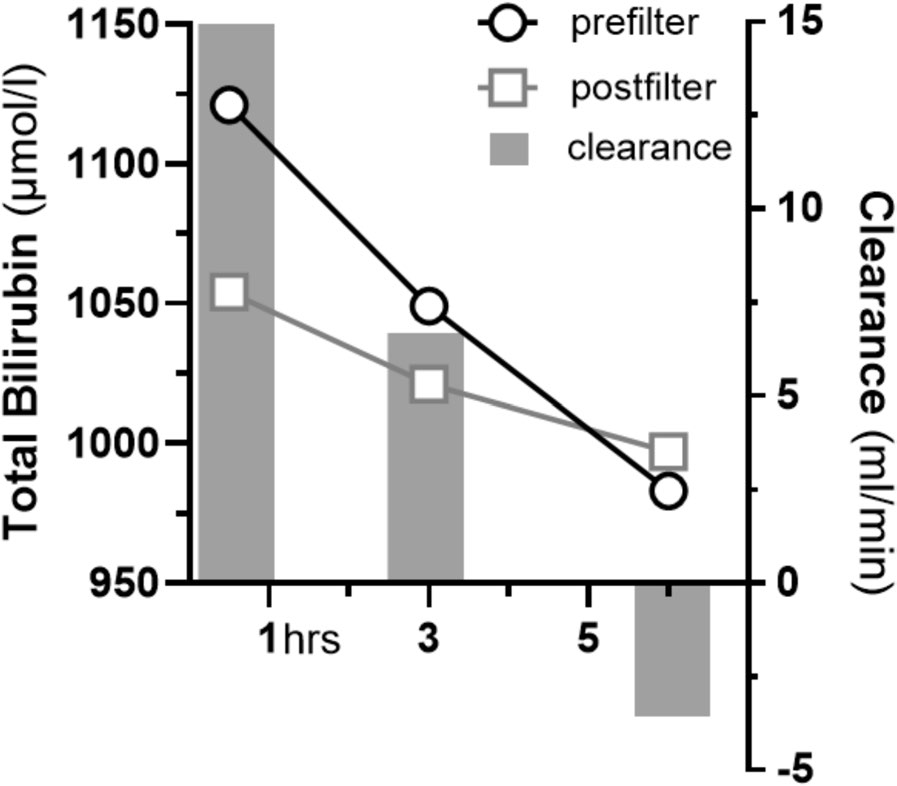
Course of total bilirubin measured pre- and postfilter during hemoperfusion and resulting clearance; Clearance rates were calculated as CI = Q × (Cpre − Cpost)/Cpre.) Q, Blood flow

One aspect that has been shown with this data set, is the declining clearance rates over time, which most likely reflects individual saturation points of the adsorber. Furthermore, for Interleukin 8 (Il-8) and Macrophage Inflammatory Protein-1 Alpha (MIP-1α) the study described a situation during the longitudinal HP treatment, in which concentrations became higher after the adsorber, representing a negative clearance or in other words a so-called desorption phenomenon. This means that cytokines were released back into the circulation by the saturated adsorber. This phenomenon has been described before for certain antibiotics [2] and MDMA [3]. In line with these observations, we found a declining and finally negative clearance rate for bilirubin in a patient with severe hyperbilirubinemia after 6 h of HP. The 28-year-old woman presented with a hemolytic crisis in Evans syndrome triggered by parainfluenza type 3 infection and developed progressive encephalopathy due to excessively elevated bilirubin. As she already received intermittent renal replacement therapy, the decision was made to add a CytoSorb^®^ adsorber to aggressively treat the hyperbilirubinemia. The clearance of bilirubin was measured longitudinally. The initial clearance of 14.9 ml/min fell rapidly over few hours and was even negative within less than 6 h (Fig. 1).

The recently published data of an ESICM survey demonstrated, that CytoSorb is the most commonly used extracorporeal blood purification technique by European intensivists [4]. Nevertheless data on how to decide on adsorber exchange intervals is scarce so far and different approaches have been described. Some suggest changing adsorbers regarding to clinical response [5]. One proposed method by Schultz et al. suggests, that the dosing of adsorption efficacy should be calculated with the amount of blood purified, but the formula does not account for adsorber changes and may be too complex for bedside application [6]. A best practice consensus from 2023 concludes, that adsorber changes should be considered every 8 to 12 h [7]. Importantly, none of the mentioned approaches considers the desorption effect and suggested time spans are longer than for the observed occurrence of a negative clearance by Jansen et al. and our observation. This is especially relevant, as Matson et al. discuss in their work hypothetical mechanisms of HP’s toxicity, one of which suggests, that the gradual desorption of cytokines may promote an inflammatory response contrary to the therapeutic intention [8].

In summary, according to the study’s data and our findings, we suggest, that adsorber change intervals should be individually evaluated. The decision could be based on the initial serum level of the substance(s) one seeks to adsorb; meaning the higher it initially is, the shorter the exchange interval should be, due to faster saturation. Ideally, measurement of serial clearance rates during course of a treatment could predict the time of adsorber saturation and indicate adsorber change. Overall desorption from extracorporeal adsorbers should be subject to further investigations.

## Data Availability

Ethics regulations do not allow data sharing from this article.

## Abbreviations

HP: Hemoperfusion
LPS: Bacterial lipopolysaccharide
Il-8: Interleukin 8
MIP-1α: Macrophage Inflammatory Protein-1 Alpha
MDMA: 3,4-Methylendioxy-*N*-methylamphetamin
